# *Pennisetum setaceum* or *Pennisetum advena* cultivars, what ornamental do we have in our garden

**DOI:** 10.1002/ece3.7908

**Published:** 2021-07-13

**Authors:** Johannes Leonardus Cornelis Hendrikus van Valkenburg, Maarten Costerus, Marcel Westenberg

**Affiliations:** ^1^ National Plant Protection Organization National Reference Centre Wageningen the Netherlands; ^2^ Plant Sciences Wageningen University & Research Wageningen the Netherlands

**Keywords:** Cenchrus, EU regulation 1043/2014, fountain grass, invasive plant, NGS

## Abstract

*Pennisetum* Rich. or following recent taxonomic insights *Cenchrus* L. is a genus with some 120 species worldwide, especially in warm areas. The genus includes some crops, some ornamentals but mostly species that are considered weedy. The name of one of the weedy species *Pennisetum setaceum* (Forssk.) Chiov. is also found on labels of ornamental grasses as *P. setaceum* “Rubrum.” It has been debated to belong to a species on its own *Pennisetum advena* Wipff & Veldkamp or *Cenchrus advena* (Wipff & Veldkamp) Morrone, only known from cultivation, whereas others still adhere to a broader species concept of *P. setaceum*. The recent inclusion of *P. setaceum* on the EU List of Union concern has revitalized the discussion on this issue for commercial reasons. Based on a morphological and molecular comparison (ITS, rbcL, and the trnh‐psbA intergenic spacer sequences) of the type specimen of *P. advena*, five of its “cultivars” in trade and collections of *P. setaceum* from different regions of the world we conclude that plants currently in trade in Western Europe belong to a separate species *P. advena*. A drooping inflorescence is consistent as is the difference in width of the leaf blade, the leaf blade being flat or involute, the central vein being swollen or not, and the length of the stipe being 0.3–1.1 mm in *P. advena* and 1.1–3.1 mm in *P. setaceum*. On the chloroplast markers rbcL and trnH‐psbA, the species consistently differ in 2 and 4 base pairs, respectively. On the nuclear ITS sequence, there is only 90% overlap between the two species. This justifies these ornamentals to be excluded from the List of Union concern of EU regulation 1143/2014.

## Graphical abstract

The proper identity of ornamental Pennisetum plants traded as “Rubrum” has been debated. The recent inclusion of *Pennisetum setaceum* on the EU list of Union concern has revitalized the discussion on this issue for commercial reasons. Based on a morphological and molecular comparison (ITS, rbcL, and the trnh‐psbA intergenic spacer sequences) of the type specimen of *Pennisetum advena*, five of its “cultivars” in trade and collections of *P. setaceum* from different regions of the world we conclude that plants currently in trade in Western Europe belong to a separate species *P. advena*.

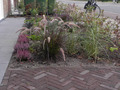

## 1 INTRODUCTION

*Pennisetum* Rich. or following recent taxonomic insights *Cenchrus* L. is a genus with some 120 species worldwide, especially in warm areas (Chemisquy et al., 2010; Kellogg, 2015). It includes some crops, some ornamentals but mostly species that are considered weedy. There has been or still is considerable confusion with respect to the proper identity of *Pennisetum* cultivars traded as, “Cherry Sparkler,” “Fireworks,” “Rubrum,” “Sky Rocket,” and “Summer Samba.”

**FIGURE 1:**
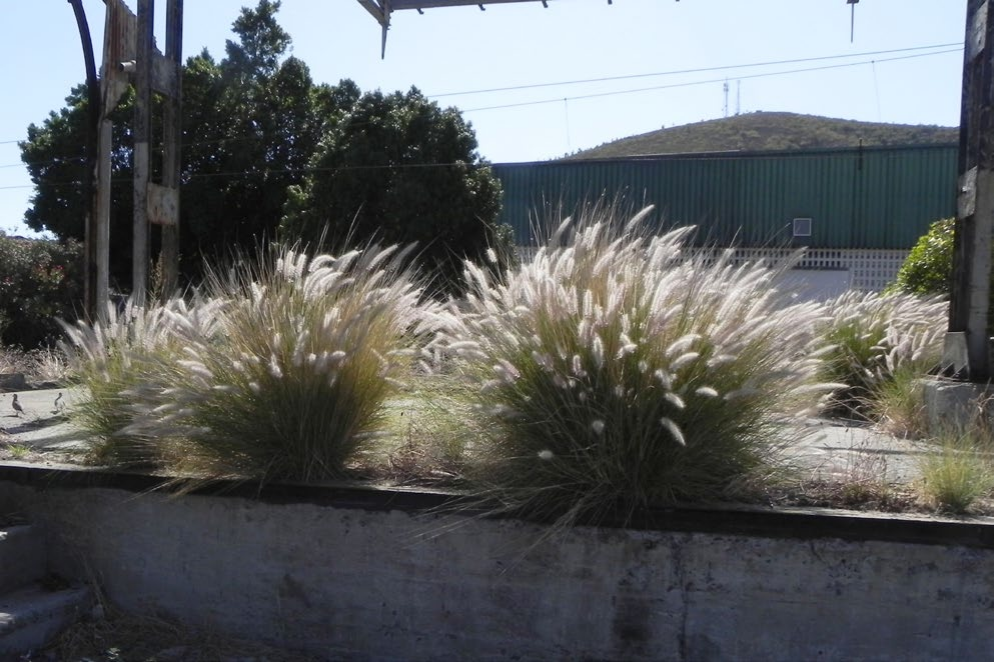
*Pennisetum setaceum* as a weed in South Africa

**FIGURE 2:**
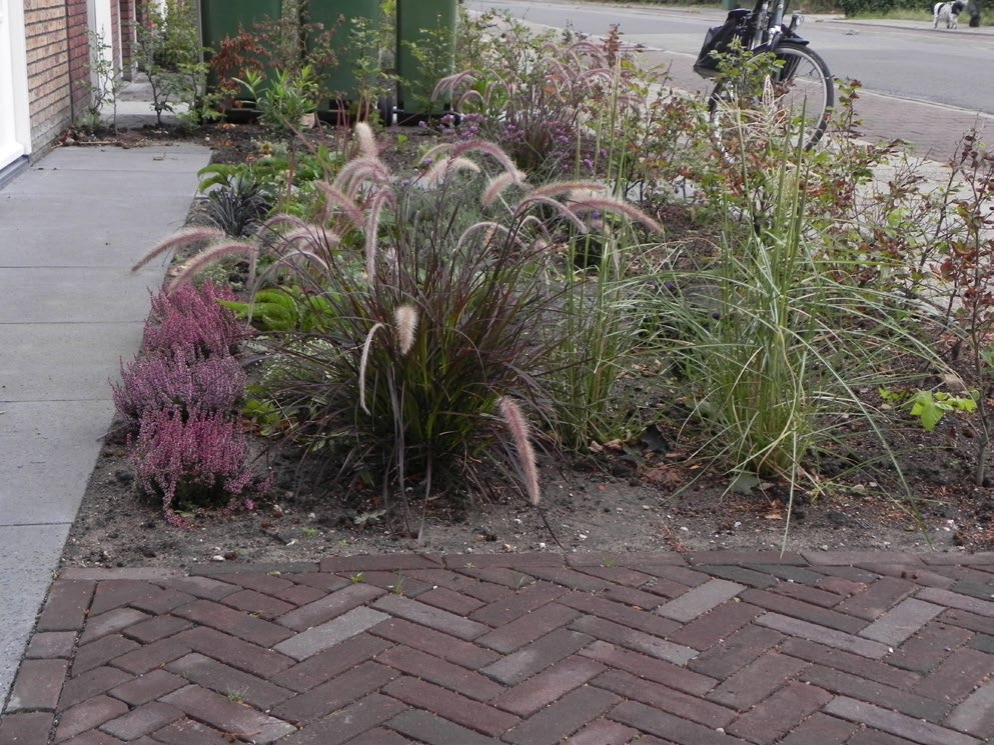
*Pennisetum advena* with characteristic drooping inflorescences in an urban garden (photographer Edu Boer)

It has been debated that these cultivars belong to a species of its own, *Pennisetum advena* Wipff and Veldkamp (1999) or *Cenchrus advena* (Wipff & Veldkamp, 1999) Morrone (Chemisquy et al., 2010; Veldkamp, 2014), only known from cultivation. However, others still adhere to a broader species concept of *Pennisetum setaceum* (Groom et al., 2017). Finally, there are those that interpret a discussion on relatedness as a proxy for a hybrid status (Meyer, 2012; Padhye et al., 2008). The recent inclusion of *P. setaceum* (Figure 1) on the List of Union concern of EU regulation 1143/2014 has revitalized the discussion on this issue for commercial reasons (see, e.g., the recommendation by Val’hor, 2017). Listing of a species on the List of Union concern implies that all its lower taxa or hybrids are potentially subject to the same rules and prohibitions, thus resulting in an effective ban on sale of these popular cultivars traded under the name of either *P. setaceum* or *P. advena* (Figure 2).

Provisional molecular results pointing to a distinction in the aforementioned cultivars (Anonymous, 2017) have resulted in these taxa being provisionally excluded from the ban, while a more detailed morphological and molecular study is conducted. Results of this study are presented here.

## 2 METHODS

### 2.1 Acquisitions of plants

For a study of *Pennisetum* species and cultivars in trade in the Netherlands, a large number of living plants was acquired from commercial growers and garden centers and subsequently grown in a quarantine glasshouse (Costerus, 2018; Costerus & van Valkenburg, 2018). In addition, a total of 168 herbarium specimen from the National Herbarium at Leiden belonging to 10 *Pennisetum* species were consulted for descriptive purposes.

For the present detailed study, plants listed in Table 1 were used. Mother plants of the five major cultivars “Cherry Sparkler,” “Fireworks,” “Rubrum,” “Sky Rocket,” and “Summer Samba” were obtained from Henk de Jong (CNB Plants) (Valkenburg 3871, 3872, 3873, 3874, 3966). The type specimen of *P. advena* (Wipff 1723) and an African collection of *P. setaceum* (Mooney 9419) were consulted at Leiden (L, WAG). Naturalized *P. setaceum* plants were collected from the Canary Islands and in Catalonia, Spain (Simons 2006, Verloove 13345, 13647, 13650). Ornamental *P. setaceum* were collected in New Zealand (Valkenburg 3934) and cuttings of *P. advena* intercepted in a mislabeled commercial import from China (Valkenburg 4026).

**TABLE 1:** *Pennisetum* specimen used in this study

Species	Collection no.	Year	Country
*P. advena*	Valkenburg 4026	2019	China
*P. advena*	Wipff 1723	1990	USA
*P. advena* “Cherry Sparkler”	Valkenburg 3874	2017	the Netherlands
*P. advena* “Fireworks”	Valkenburg 3873	2017	the Netherlands
*P. advena* “Rubrum”	Valkenburg 3966	2018	the Netherlands
*P. advena* “Sky Rocket”	Valkenburg 3872	2017	the Netherlands
*P. advena* “Summer Samba”	Valkenburg 3871	2017	the Netherlands
*P. setaceum*	Mooney 9419	1962	Eritrea
*P. setaceum*	Simons 2006	2017	Canary Islands (Spain)
*P. setaceum*	Valkenburg 3934	2018	New Zealand
*P. setaceum*	Verloove 13345	2018	Spain
*P. setaceum*	Verloove 13647	2019	Spain
*P. setaceum*	Verloove 13650	2019	Spain

### 2.2 Macromorphological approach

Of all living material height of the plant including inflorescence was measured, color of the stem, width, length and color of leaves; color and length of inflorescence; length of spikelet; texture of axis of inflorescence; length of stipe; number of spikelets per fascicle; and color, length and presence of long hairs on bristles. For all herbarium specimens, macromorphological measurements were similar. All measurements are used to build an interactive image‐driven key using LUCID software (Identic, Stafford Heights, Australia).

### 2.3 DNA extractions

Genomic DNA was isolated from approximately 100 mg plant material with the DNeasy Plant Mini Kit (Qiagen, Venlo, the Netherlands) using the TissueLyser procedure and eluted with 50 μl prewarmed (65°C) AE buffer. DNA was stored at −20°C until use.

### 2.4 PCR amplification and Sanger sequencing

PCRs for the chloroplast rbcL gene and trnH‐psbA intergenic spacer and nuclear ITS (partial 18S, ITS1, 5.8S, ITS2, partial 28S) loci were performed in 25 μl reaction mixes containing 200 nmol/L of either primers rbcL‐a F and rbcLa SI_Rev, trnH2, and psbAF or ITS5 and ITS4 (Table 2), respectively, 1 x MyFi^TM^ Mix (Bio‐line, Taunton, USA) and 2 μl genomic DNA. The cycle conditions for rbcL and trnH‐psbA loci were as follows: 5 min at 95°C, followed by 5 cycles of 30 s at 94°C, 30 s at 45°C, 30 s at 72°C and 35 cycles of 30 s at 94°C, 30 s at 50°C, 30 s at 72°C and a final extension for 10 min at 72°C. The cycle condition for ITS locus was as follows: 5 min at 95°C, followed by 40 cycles of 30 s at 94°C, 30 s at 52°C, 100 s at 72°C, and a final extension for 10 min at 72°C.

**TABLE 2:** Primers used in this study

loci	Primer name	Primer sequence	Reference
rbcL	rbcL‐a F	ATGTCACCACAAACAGAGACTAAAGC	Kress & Erickson, (2007)
	rbcLa SI_Rev	GTAAAATCAAGTCCACCRCG	Kress et al. (2009)
trnH‐psbA	trnH2	CGCGCATGGTGGATTCACAATCC	Tate, (2002)
	psbAF	GTTATGCATGAACGTAATGCTC	Sang et al. (1997)
ITS	ITS5	GGAAGTAAAAGTCGTAACAAGG	White et al. (1990)
	ITS4	TCCTCCGCTTATTGATATGC	White et al. (1990)

PCR products were purified using the QIAquick PCR Purification Kit (Qiagen, Venlo, the Netherlands) preceding bidirectional cycle sequencing with the BigDye Terminator v1.1 Cycle Sequencing Kit (Thermo Fisher Scientific, Bleiswijk, the Netherlands) using amplification primers as sequencing primers in separate reactions according to the manufacturer's instructions. Cycle sequence products were purified with the DyeEx 2.0 Spin Kit (Qiagen, Venlo, the Netherlands) and sequenced using a 3500 Genetic Analyzer (Thermo Fisher Scientific, Bleiswijk, the Netherlands). Consensus sequences were generated from an assembly with trace files from both Sanger sequencing runs in Geneious R10 (Biomatters Auckland, New Zealand). Amplification primer sequences were trimmed in the assembly, and when needed, additional trimming was performed to obtain high‐quality (PHRED >30) consensus sequences.

### 2.5 Illumina sequencing

Genomic DNA was Illumina sequenced (PE150) with the NextSeq 500 V2 platform with minimal 2 Gb output per sample. The NEBNext^®^ Ultra DNA Library Prep Kit for Illumina (New England Biolabs, Ipswich, USA) was used to process the samples. Fragmentation of the DNA using the Bioruptor Pico (Diagenode, Liège, Belgium), ligation of sequencing adapters, and PCR amplification of the resulting product were performed according to the procedure described in the NEBNext Ultra DNA Library Prep Kit for Illumina Instruction Manual. Reads were reference assemble to the rbcL, trnH‐psbA spacer, and ITS sequences of a *Pennisetum orientale* specimen (NPPO‐NL 6148331) using CLC genomic workbench v10 (Qiagen) with default settings. Variants were detected by using the Basic Variant Detection tool in CLC genomic workbench v10 with default settings.

## 3 RESULTS

### 3.1 Macromorphological differences observed

The analysis of all living material and the herbarium collections of the wild samples and cultivars belonging to the 10 species can be found in Costerus (2018) and Costerus and van Valkenburg (2018). It has been reworked to an interactive image‐driven identification key using LUCID software and can be found at https://keys.lucidcentral.org/keys/v3/pennisetum/en/index.html.

For this paper, we focus on the distinguishing characters between *P. advena* and *P. setaceum*.

Most obvious morphological characters when judging its ornamental value are the culm of *P. setaceum* being rarely branched as opposed to *P. advena* and the inflorescence of the latter being flexuous and drooping as opposed to rigid in *P. setaceum*. Both characters are added to the attractiveness of the species. Leaf characters in addition to the color of *P. setaceum* never being purplish are the consistently larger width and flatness of the blade in *P. advena* as well as the more prominent thickened central vein and involute leaf in *P. setaceum*. These character differences also apply to the ornamental *P. setaceum* collected in New Zealand. More detailed measurements on the inflorescence and leaves of *P. setaceum*. *P. advena* and cultivars are given in Table 3 with the values as published for both species (Veldkamp, 2014) for comparison.

**TABLE 3:** Distinguishing macromorphological characters of the type specimen of *Pennisetum advena* (J.K. Wipff 1723 L), the five cultivars “Cherry Sparkler” (Valkenburg 3874), “Fireworks” (Valkenburg 3873), “Rubrum” (Valkenburg 3966), “Sky Rocket” (Valkenburg 3872), and “Summer Samba” (Valkenburg 3871), *P. setaceum* culta in New Zealand (Valkenburg 3934) and values for both species in Veldkamp (2014)

	*P. advena*	Cherry Sparkler	Fireworks	Rubrum	Sky Rocket	Summer Samba	*P. setaceum*	*P. advena* (Veldkamp)	*P. setaceum* (Veldkamp)
	Wipff 1723	Valkenburg 3874	Valkenburg 3873	Valkenburg 3966	Valkenburg 3872	Valkenburg 3871	Valkenburg 3934		
Leaf width (mm)	7–9	4–6.5	3–8.5	2–4	3–5.5	4–6	1–2	6–11	1–3.7
Leaf length (cm)	50	36–42	28–49	20–46	20–41	31–44	15–27	22–52	30–100
Leaf color	Green, purple	Green, purple, white	Green, purple, white	Green, purple	Green, white	Green, purple, white	Green	Green, purple,	Green
Length of inflorescence (cm)	21–27	21	16–23	15–22	17–18	18–22	17	22–32	10–26
Length of fascicle (mm)	5.2–5.8	4–5	4–6	3–5	4–6.5	4–5	5.5–6.2	5.2–6.5	4.7–6.8
Length of stipe (mm)	0.44–0.82	0.6–0.9	0.3–0.6	0.4–0.5	0.7–1.0	0.4–0.5	1.7–1.8	0.5–1.1	1–3
Length of longest bristle (mm)	30–37	23–32	22–29	25–33	22–34	25–33	28–32	21–33.5	28–32

### 3.2 Sequence analysis

The results of the sequence analysis for the chloroplast rbcL gene and trnH‐psbA intergenic spacer as well as for the nuclear ITS (partial 18S, ITS1, 5.8S, ITS2, partial 28S) sequence are summarized in Table 4. Based on the rbcL and trnH‐psbA sequences, *P. advena* and *P. setaceum* can be distinguished from each other. For rbcL, there are two nucleotide (nt) differences, and for trnH‐psbA region, there are four nt differences between *P. advena* and *P. setaceum*. All five cultivars could be matched to the type specimen of *P. advena*. Interestingly, the trnH‐psbA region of *P. setaceum* sample “Simons 2006” could not be bidirectionally sequenced by Sanger technology. Illumina sequence data revealed that this problem was caused by the presence of two different variants with a single indel. Each of these variants was present in equal amounts.

**TABLE 4:** Sequencing results of the rbcL, trnh‐psbA, and ITS loci for the *Pennisetum* specimen

Species	collection no.	Sequencing	rbcL	trnH‐psbA	ITS
*P. advena*	Valkenburg 4026	S^1^	a^3^	a	aI^7^, aII^8^
*P. advena*	Wipff 1723	S, I^2^	a	a	aI, aII
“Cherry Sparkler”	Valkenburg 3874	S, I	a	a	aI, aII, sI
“Fireworks”	Valkenburg 3873	S, I	a	a	aI, aII
“Rubrum”	Valkenburg 3966	S, I	a	a	aI, aII
“Sky Rocket”	Valkenburg 3872	S, I	a	a	aI, aII, sI
“Summer Samba”	Valkenburg 3871	S, I	a	a	aI, aII
*P. setaceum*	Mooney 9419	I	s^4^	s	sI, sII
*P. setaceum*	Simons 2006	S, I	s	sI^5^, sII^6^	sI, sII
*P. setaceum*	Valkenburg 3934	S	s	s	sI, sII
*P. setaceum*	Verloove 13345	S	s	s	sI, sII
*P. setaceum*	Verloove 13647	S	s	s	sI, sII
*P. setaceum*	Verloove 13650	S	s	s	sI, sII

^1^ S = Sanger sequencing.

^2^ I = Illumina sequencing.

^3^ a = *Pennisetum advena* sequence.

^4^ s = *Pennisetum setaceum* sequence.

^5^ sI = *Pennisetum setaceum* variant I sequence.

^6^ sI = *Pennisetum setaceum* variant II sequence.

^7^ aI = *Pennisetum advena* variant I sequence.

^8^ aII = *Pennisetum advena* variant II sequence.

Sanger sequencing of the ITS region revealed the presence of SNPs in both *P. advena* (*n* = 3) and *P. setaceum* (*n* = 2) specimens. Illumina sequencing revealed that in each of the specimens, two variants of the ITS sequence were present. For *P. advena,* the ratio between these two variants was for each specimen approximately 55% for variant aI and 45% for variant aII. For *P. setaceum* sample “Simons 2006,” the ratio between variants sI and sII was approximately 70% and 30%, while for *P. setaceum* sample “Mooney 9419,” this was the other way around (30% sI and 70% sII). Nevertheless, *P. advena* and *P. setaceum* could be distinguished based on their ITS sequences as they are only 90% identical. The cultivars “Fireworks,” “Rubrum,” and “Summer Samba” contained both variants I and II of the *P. advena* ITS sequences. Based on all three loci (rbcL, trnH‐psbA, and ITS), these three cultivars could therefore be matched to *P. advena*.

The ITS sequences of the cultivars “Cherry Sparkler” and “Sky Rocket” could not be obtained by Sanger sequencing. Illumina sequencing of these cultivars revealed that both cultivars contained ITS variants I and II of *P. advena* (aI and aII) and variant I of *P. setaceum* (sI). For “Cherry Sparkler,” the ratio between the different variants was approx. 45% (aI), 15% (aII), and 40% (sI) and for “Sky Rocket” 20% (aI), 50% (aII), and 30% (sI).

## 4 DISCUSSION

From a scientific point of view, the above‐mentioned *Pennisetum advena* and *P. setaceum* should be addressed as *Cenchrus* species, following recent molecular and phylogenetic studies on the genera *Cenchrus* and *Pennisetum* (Chemisquy et al., 2010; Donadio et al., 2009). Numerous publications have already made the new necessary combinations for the American (Chemisquy et al., 2010), European (Verloove, 2012; Verloove et al., 2014), Southeast Asian (Veldkamp, 2014), Australian (Symon, 2010), and Pacific species (Tornabene & Wagner, 2013). However, as the ornamental plant trade is rather conservative, we have retained the old *Pennisetum* names for *P. advena* and *P. setaceum* and the other common ornamentals *P. orientale* and especially *P. alopecuroides* both represented by numerous cultivars in trade. Likewise, the name *Pennisetum* has been retained on the EU List of Union concern based on the risk assessment by Danas and Verloove (2015).

Although the exact origin of the ornamental plants commonly referred as *P. setaceum* “Rubrum” is unclear, apart from it being Old World, its introduction in the United States in 1916 is well documented as well as the quest for a valid name for the species. For an elaborate discussion, see Wipff and Veldkamp (1999).

Confusion on hybrid status goes back to Padhye et al. (2008) who somehow misinterpreted the Wipff and Veldkamp (1999) discussion on the origins of *P*. *advena*. This misinterpretation was later repeated by Meyer (2012) and has been adopted by numerous people who apparently failed to verify this in the original publication by Wipff and Veldkamp (1999). This view has been expressed already by Melanie Schori from USDA (pers. comm. 2017) in response to questions raised by European umbrella organizations of plant growers to the Community Plant Variety Office, following listing of *P. setaceum* on the List of Union concern.

Descriptions of some of the patented cultivars such as “Fireworks,” “Cherry Sparkler,” and “Sky Rocket” can be found online as it applies to the United States. Within a European context, as a different legislation applies reference can be made to the website of the Community Plant Variety Office (https://cpvoextranet.cpvo.europa.eu/mypvr/#!/en/publicsearch). Descriptive aspects and a comprehensive bibliography of relevant cultivars are already dealt with in the recommendation for *Pennisetum* by Val’Hor (2017) and by Costerus (2018).

Distinguishing morphological characters for *P. advena* have been elaborately discussed by Wipff and Veldkamp (1999). Some refinements resulting from our study can be added. Branching in aerial nodes does sometimes occur in *P. setaceum* as observed in cultivated plant in New Zealand (Valkenburg 3934) and wild plants in Ethiopia (P.C.M. Jansen 3946). Drooping inflorescence is consistent as is the difference in width of the leaf blade, the leaf blade being flat or involute, the central vein being swollen or not, and the length of the stipe being 0.3–1.1 mm in *P. advena* and 1.1–3.1 mm in *P. setaceum*.

From an European perspective, a difference in potential risk of invasiveness of *P. advena* compared with *P. setaceum* would be a major criterion to justify it not to be regulated. Simpson and Bashaw (1969) showed that *P. advena* (purple *P. setaceum*) rarely sets seed under field conditions and does not behave as an apomictic species nor is the plant capable of selfing as opposed to *P. setaceum*. Chilling injury studies point to hardiness issues for *P. advena* when temperatures drop below 5°C for 2 weeks (Padhye et al., 2008). In addition, there are no records of invasive behavior of “purple” *P. setaceum*.

Sequences from the chloroplast loci rbcL and trH‐psbA as well as the nuclear loci ITS showed enough resolution to distinguish *P. advena* from *P. setaceum*. However, Blast searches in the NCBI GenBank with the rbcL and trnH‐psbA sequences revealed very high identities (up to 100%) with other *Pennisetum*, *Cenchrus,* and *Setaria* species, which makes identification based on these two loci alone difficult. Furthermore, GenBank contains a few accessions for *P. setaceum* (or *Cenchrus setaceus*), but none for *P. advena*. In addition, the rbcL and trnH‐psbA sequences (GenBank acc. GU135184 and GU135350) for the *Cenchrus setaceus* voucher J.R. Abbott 24732 (FLAS) are identical to the rbcl and trnH‐psbA sequences of *P*. *advena* from this study, suggesting a misidentification.

To obtain more resolution for molecular identification, the sequence of the nuclear ITS locus was used. Interestingly, both *P. advena* and *P. setaceum* each contained two variants of the ITS sequences with 2 and 3 SNP, respectively. Most likely these variants are caused by their polyploid nature, as *P. setaceum* for instance is known to be hexaploid (Martel et al., 2004), although plants in general may contain multiple copies of ITS (Feliner & Rosselló, 2007). The ratio between the two variants was not the same in each of the specimens, but this could be caused by a sequence artifact or an uneven distribution of the variants among the different chromosome copies.

Using the ITS sequence as a proxy for species hybridization has been successfully used for the identification of *Impatiens* hybrids (van Valkenburg et al., 2019). The cultivars “Cherry Sparkler” and “Sky Rocket” contained the ITS sequences from both *P. advena* and *P. setaceum*, suggesting that these specimens have a hybrid status. Interesting, they contain both *P. advena* ITS variants, but only one of the *P. setaceum* variants. This points to a hybrid origin, with *P. advena* being the seed plant.

## 5 CONCLUSION

Based on molecular and morphological characters, both species can be clearly separated and the popular ornamentals in trade in Europe belong to *P. advena*. This justifies these ornamentals to be excluded from the List of Union concern of EU regulation 1143/2014.

## CONFLICT OF INTEREST

The authors have no conflict of interest to report.

## AUTHOR CONTRIBUTIONS

**Johannes Leonardus Cornelis Hendrikus van Valkenburg:** Conceptualization (lead); Data curation (equal); Formal analysis (supporting); Investigation (equal); Supervision (lead); Writing‐original draft (equal); Writing‐review & editing (equal). **Maarten Costerus:** Conceptualization (equal); Data curation (equal); Formal analysis (lead); Writing‐original draft (equal); Writing‐review & editing (equal). **Marcel Westenberg:** Data curation (equal); Formal analysis (equal); Writing‐original draft (equal); Writing‐review & editing (equal).

## Data Availability

DNA sequences: ITS, rbcL, and the trnh‐psbA intergenic spacer sequences were deposited in NCBI GenBank under the following accession numbers: MW177954‐MW178003. Sequences will also be made available at https://qbank.eppo.int/plants/. Herbarium vouchers are available at BR, L, WAG, WAG‐PD, and online at https://www.q‐bankplants.eu/.
